# Medical Statistics in the Army

**Published:** 1867-06

**Authors:** 


### MEDICAL STATISTICS IN THE ARMY.

Dr. Benjamin Howard, of New York, offered the following preamble and resolutions:

*Whereas,* There has been issued, and still remains in force, an official order from the Surgeon General of the United States Army prohibiting the communication of any medical or surgicial information, by any medical officer of the United States Army, to any person whatsoever, without special permission from the Medical Bureau at Washington; thus appropriating, as far as the official power of the Surgeon General can compass it, all the valuable experience and statistics of all medical men who have served in the various departments of the United States Army to the exclusive use of the Medical Bureau; and,

*Whereas,* Under such arbitrary control an official report has already been made tending to create incorrect impressions on scientific questions of great practical importance to the profession and to society; and,

*Whereas,* It is important to the reputation of all men, who served during the war, that they have the opportunity of correcting such erroneous impressions by an examination of the original records; therefore, be it

*Resolved,* That it is the opinion of this Association that the monopoly now exercised by the Medical Bureau over the medical and surgicial records of the war, is contrary to the genius and catholic spirit of our profession, and obstructive to the highest interests of science and humanity.

*Resolved,* That the Secretary of War, or other proper authorities, be requested to direct that the original records of the medical and surgicial history of the war be rendered accessible, on certain regular days of each month, for purposes of scientific investigation, to all medical men who have served as such in the army of the United States.

Dr. Woodward, of the U. S. Army, and occupying some position in the Surgeon General's office, replied curtly to the proposition and speech of Dr. Howard, charging him with ventilating his private griefs in such a way as to reflect upon the honest and faithful performance of duty by Government officers. The simple fact was, that in the routine of duty of the office there were fifty clerks engaged in examining the reports referred to, investigating the claims of officers, soldiers, and widows of the same, for pensions, etc., and in making up the medical report for publication. To admit any portion of the public to these documents would retard the performance of duty toward these needy claimants. He therefore moved to lay the preamble and resolutions on the table, which was agreed to almost unanimously.

